# Retraction: Endocytic recycling and vesicular transport systems mediatetranscytosis of Leptospira interrogans across cell monolayer

**DOI:** 10.7554/eLife.65227

**Published:** 2020-12-03

**Authors:** Yang Li, Kai-Xuan Li, Wei-Lin Hu, David M Ojcius, Jia-Qi Fang, Shi-Jun Li, Xu'ai Lin, Jie Yan

Li Y, Li K-X, Hu W-L, Ojcius DM, Fang J-Q, Li S-J, Lin X, Yan J. 2019. Endocytic recycling and vesicular transport systems mediate transcytosis of Leptospira interrogans across cell monolayer. *eLife* **8**:e44594. doi: 10.7554/eLife.44594.

Published 23, April 2019

The authors are retracting this article in response to concerns around image presentation that were first raised on PubPeer on August 20, 2020. We investigated this matter and examined the figures in this paper carefully. Out of more than 2,000 images in the article, six of them were reused mistakenly and unintentionally:

Figure 2B contains two duplicated Western blot bands; one of the transmission electron microscopy images from Figure 1C (BJ panel) overlaps with an image published in Figure 1B from our lab in 2018 (Emerging Microbes & Infections, https://doi.org/10.1038/s41426-018-0135-9); and there are three duplicated confocal images within Figure 4–figure supplement 1 (panel D).

As our request for a Correction was not accepted by the editors, the following authors have agreed to the retraction: Yang Li, Kai-Xuan Li, Wei-Lin Hu, David M Ojcius, Shi-Jun Li, Xu'ai Lin, Jie Yan. We also contacted the following author about the retraction but did not receive a response: Jia-Qi Fang.

